# Reply to Sagliocco, O.; Betelli, M. Comment on “Fierro et al. Severe Hypotension, Bradycardia and Asystole after Sugammadex Administration in an Elderly Patient. *Medicina* 2021, *57*, 79”

**DOI:** 10.3390/medicina58010136

**Published:** 2022-01-17

**Authors:** Carmen Fierro, Alessandro Medoro, Donatella Mignogna, Carola Porcile, Silvia Ciampi, Emanuele Foderà, Romeo Flocco, Claudio Russo, Gennaro Martucci

**Affiliations:** 1 Department of Medicine and Health Sciences, University of Molise, 86100 Campobasso, Italy; c.fierro@studenti.unimol.it (C.F.); alessandro.medoro@unimol.it (A.M.); donatella.mignogna@studenti.unimol.it (D.M.); carola.porcile@unimol.it (C.P.); s.ciampi@studenti.unimol.it (S.C.); e.fodera@studenti.unimol.it (E.F.); 2 Anesthesia and Resuscitation Unit, “A. Cardarelli” Hospital, Azienda Sanitaria Regionale del Molise, 86100 Campobasso, Italy; romeoflocco@hotmail.com (R.F.); gennaromart@libero.it (G.M.); 3 UOC Governance del Farmaco, Azienda Sanitaria Regionale del Molise, 86100 Campobasso, Italy

We thank Dr. Sagliocco and Dr. Betelli for their comments [1] on our recent case report entitled “Severe Hypotension, Bradycardia and Asystole after Sugammadex Administration in an Elderly Patient” [2]. Sagliocco and Betelli proposed that a diagnosis of Kounis or Kounis-like syndrome could be strongly considered in our published case report, underlying the risk of underestimation of this syndrome.

Kounis syndrome is defined as “the concurrence of acute coronary syndromes, including coronary spasm, acute myocardial infarction, and stent thrombosis, with conditions associated with mast-cell and platelet activation and involving interrelated and interacting inflammatory cells, such as macrophages and T-lymphocytes, in the setting of allergic or hypersensitivity and anaphylactic or anaphylactoid insults” [3]. So far, three variants of Kounis syndrome have been described: the type I variant is characterized by the release of inflammatory mediators that induce coronary artery spasm with or without increasing cardiac enzymes and troponins; in the type II variant, the release of inflammatory mediators can induce coronary artery spasm with plaque erosion or rupture and manifests as acute myocardial infarction; patients with the type III variant are characterized by coronary artery stent thrombosis as a consequence of an allergic reaction [4].

On the one hand, we cannot exclude anaphylaxis as the primary cause of cardiac arrest, especially considering the absence of information about tryptase level or subsequent allergy testing with sugammadex, even if anaphylaxis lab tests could be misleading in the evaluation of sugammadex-induced severe anaphylaxis [5]. We also do not dispute that bradycardia could be a symptom of anaphylaxis; however, our patient responded to a single dose of ephedrine and atropine without a relapse of hemodynamic instability and there was no need to dispense boluses or an epinephrine drip, thus suggesting a direct cardiovascular effect rather than effect secondary to anaphylaxis [6].

In detail and as reported by Abdelghany et al. [4], only 1.7% of patients with Kounis syndrome show normal ECG manifestations without ST elevation or depression and 60.6% show elevated troponins. Therefore, in the case described by Yanai and Ariyoshi [7], in which two cardiac arrests in the same patient are induced after sugammadex administration five months apart each other, sinus tachycardia without specific ST segment elevation or depression and with elevated troponin I level appeared after the first cardiac arrest, while diffuse ST depression with an elevated troponin I level appeared in the second cardiac arrest. Interestingly, these two very common and characteristic features of Kounis syndrome (abnormal ECG manifestations and elevated troponin levels) were not present in our patient. Although our patient was not subjected to coronary angiography, a diagnostic tool that is useful for the coronary spasm detection, single-photon emission computer tomography has recently been used in the diagnosis of type I Kounis syndrome showing severe myocardial ischemia, while coronary angiography showed normal coronary arteries [8].

Furthermore, allergic manifestations such as skin rash, erythema, hives, and wheezes are often described during Kounis syndrome [4], symptoms that are all present in the fifteen cases of hypersensitivity reactions after sugammadex administration reviewed by Tsur and Kalansky [9].

Moreover, the first case of cardiac arrest following sugammadex-induced anaphylaxis described in 2018 by Obara et al. [10] is different from our case for two main reasons: (a) the patient described by Obara et al. was on medication for chronic paroxysmal atrial fibrillation, while our patient, despite his advanced age, had no medical history of cardiovascular disease before the administration of sugammadex; (b) our patient showed severe bradycardia one minute after sugammadex administration, and its hemodynamics rapidly improved with atropine and ephedrine and did not show any electrocardiographic signs of cardiac ischemia or arrhythmia, while the patient described by Obara et al. showed marked hypotension six minutes after the administration of sugammadex and was first treated with fluid and 0.1 mg phenylephrine, subsequently showing ST depression and polymorphic ventricular premature contraction that were treated with additional phenylephrine (0.1 mg) and epinephrine (0.1 mg). However, the patient went into cardiac arrest fourteen minutes after sugammadex administration and, even though he was treated with intravenous boluses of epinephrine (1 × 3 mg), 1 g methylprednisolone, 50 mg hydroxyzine hydrochloride, and a large volume of fluids, he recovered his systolic blood pressure and sinus heart rhythm after 10 minutes of cardio-pulmonary resuscitation.

Regarding the therapeutic management of Kounis syndrome in patients with the type I variant, treatment of the allergic event alone (intravenous corticosteroids, calcium channel blockers, and nitrates) can abolish symptoms, while in patients with the type II variant, treatment should be initiated as it would be for an acute coronary event protocol followed by corticosteroids and antihistamines [3]. However, none of these drugs were used to treat our patient, while the administration of an anticholinergic agent such as atropine to treat bradycardia induced by sugammadex, as suggested by the technical information sheet [11], and ephedrine to treat hypotension restored spontaneous cardiac activity and hemodynamic stability very quickly.

In conclusion, our feeling is that Kounis syndrome can still be excluded from the possible causes of hypotension, bradycardia, and asystole in our case report. However, as also supported by the comment by Sagliocco and Betelli together with the numerous reports of adverse cardiovascular events after sugammadex administration, it is necessary to gain insight into the different molecular and physiological changes induced by sugammadex in these patients in order to make an accurate and prompt diagnosis and, accordingly, to carry out the right pharmacological approach, considering, to date, the difficulty in forming differential diagnosis and the lack of therapeutical guidelines. 
